# Discrepancies between the medical record and the reports of patients with acute coronary syndrome regarding important aspects of the medical history

**DOI:** 10.1186/1472-6963-12-78

**Published:** 2012-03-26

**Authors:** Chete Eze-Nliam, Kellie Cain, Kasey Bond, Keith Forlenza, Rachel Jankowski, Gina Magyar-Russell, Gayane Yenokyan, Roy C Ziegelstein

**Affiliations:** 1 Division of Hospital Medicine, Johns Hopkins Bayview Medical Center, Baltimore, MD, USA; 2 Department of Psychiatry and Behavioral Sciences, Johns Hopkins University School of Medicine, Baltimore, MD, USA; 3 Department of Psychology, Loyola University Maryland, Baltimore, MD, USA; 4 Department of Pastoral Counseling, Loyola University Maryland, Baltimore, MD, USA; 5 Johns Hopkins Biostatistics Center, Johns Hopkins University Bloomberg School of Public Health, Baltimore, MD, USA; 6 Department of Medicine, Johns Hopkins University School of Medicine, Baltimore, MD, USA

## Abstract

**Background:**

Many critical treatment decisions are based on the medical history of patients with an acute coronary syndrome (ACS). Discrepancies between the medical history documented by a health professional and the patient's own report may therefore have important health consequences.

**Methods:**

Medical histories of 117 patients with an ACS were documented. A questionnaire assessing the patient's health history was then completed by 62 eligible patients. Information about 13 health conditions with relevance to ACS management was obtained from the questionnaire and the medical record. Concordance between these two sources and reasons for discordance were identified.

**Results:**

There was significant variation in agreement, from very poor in angina (kappa < 0) to almost perfect in diabetes (kappa = 0.94). Agreement was substantial in cerebrovascular accident (kappa = 0.76) and hypertension (kappa = 0.73); moderate in cocaine use (kappa = 0.54), smoking (kappa = 0.46), kidney disease (kappa = 0.52) and congestive heart failure (kappa = 0.54); and fair in arrhythmia (kappa = 0.37), myocardial infarction (kappa = 0.31), other cardiovascular diseases (kappa = 0.37) and bronchitis/pneumonia (kappa = 0.31). The odds of agreement was 42% higher among individuals with at least some college education (OR = 1.42; 95% CI, 1.00 - 2.01, p = 0.053). Listing of a condition in medical record but not in the questionnaire was a common cause of discordance.

**Conclusion:**

Discrepancies in aspects of the medical history may have important effects on the care of ACS patients. Future research focused on identifying the most effective and efficient means to obtain accurate health information may improve ACS patient care quality and safety.

## Background

Medical errors contribute significantly to morbidity and mortality [1]. Studies have shown that at least 44,000 Americans die yearly as a result of medical errors [2,3]. Total national costs (lost income, lost household production, disability and health care costs) of medical errors are estimated to be nearly $1 billion dollars, a significant amount due to associated health care costs [4]. Common causes of medical errors include adverse drug events [5], wrong identification of patients or site of procedure [6], and poor communication between health care givers and patients [7]. Medical records have also been shown to sometimes contain inaccuracies [8,9]. Medication reconciliation, which involves obtaining and documenting a list of medications from the patient and ensuring that this matches the list in the medical record, has been established as a Joint Commission National Patient Safety Goal [10]. Without a similar process of health history reconciliation, however, many important discrepancies may exist that can affect the care of hospitalized patients. This may be particularly relevant to the care of a patient with an acute myocardial infarction or unstable angina pectoris (often referred to as "acute coronary syndrome or ACS") since important ACS treatment decisions are based on the medical history. Treatment of ACS is guided by early risk stratification of the patients, using specially-developed risk scores that typically use the patient's self-report (e.g., of angina), to decide whether early interventional therapy should be pursued [11]. While there have been studies which evaluated concordance between self-report and medical records in cardiovascular care patients including patients with myocardial infarction [12,13], to the best of our knowledge, this is the only study where self-report and medical records were obtained nearly concurrently, thereby significantly decreasing problems with recall with the potential of yielding more objective estimates. Therefore, the aim of this study was to investigate the degree of agreement and sources of non-agreement between the documentation of ACS patients' medical history as part of usual care and the patient's self-reported health history during the same hospitalization.

## Methods

This study was cross-sectional in design. Individuals 18 years of age and older with a diagnosis of ACS were eligible for participation if they were admitted between August 2008 and June 2009 to the Cardiology service of Johns Hopkins Bayview Medical Center, a 560-bed teaching hospital of the Johns Hopkins Health System. The diagnosis of ACS was based on the presence of symptoms consistent with acute myocardial ischemia within 24 h of presentation and at least one of the following: documented coronary artery disease, ischemic changes on the electrocardiogram or elevated levels of cardiac troponin I. Individuals with ACS were invited to participate in the study during hospitalization after obtaining informed consent. Exclusion criteria were inability to complete questionnaires, presence of cocaine or other recreational stimulants on admission toxicology screen, intubation or sedation more than three days after hospitalization, and dementia precluding the ability to provide informed consent or reliably complete questionnaires. The study was approved by the Johns Hopkins Institutional Review Board.

A health history questionnaire (HHQ) that inquired about the presence of different health conditions was administered to the participants. The HHQ documented self-reported information about demographics (age, gender, occupation, race and ethnicity, education) as well as the presence of 40 general medical conditions, 9 mental health conditions, and prior surgical procedures. The questionnaire has been used in other clinical studies [14,15]. For the present analysis, 13 conditions from the HHQ, selected a priori by the authors CME and RCZ, considering their potential to influence treatment choice of the ACS patients, were angina; arrhythmia; myocardial infarction; congestive heart failure; hypertension; other cardiovascular diseases which include peripheral vascular disease and hyperlipidemia; diabetes mellitus; cerebrovascular accident; chronic kidney disease; cocaine use; bronchitis or pneumonia; chronic obstructive pulmonary disease; and cigarette smoking. In the HHQ, patients were asked whether they have had a particular condition in the past, at present or never. To facilitate comprehension of the terms on the HHQ, non-medical terms were used to describe these conditions where appropriate; for instance patients were told angina was analogous to chest pain and arrhythmia to an irregular heartbeat. Subjects also completed the mini-mental state examination (MMSE). The MMSE is a test that has been shown to discriminate between patients with and without cognitive impairment with scores of 20 or less found only in patients with dementia, schizophrenia, delirium and affective disorder, and has demonstrated satisfactory reliability and construct validity [16]. Participants were allowed to complete questionnaires at their convenience. All subjects completed questionnaires within 24 h of receiving them and prior to hospital discharge.

The electronic medical records were reviewed and the most detailed admission notes examined for information related to patient demographics (age, gender, occupation, race and ethnicity, education) and medical, surgical, and mental health conditions. Where no admission note was entered in the electronic medical record, the written medical record was reviewed to obtain this information. Data from the HHQ and medical record relating to patient demographics and to the aforementioned 13 conditions were entered into a Microsoft Excel spreadsheet and coded appropriately. The data from the medical record for angina, smoking and cocaine (past, present and never) condition were compared with the data from HHQ (past, present, never). The presence or absence of these conditions was noted according to the patients' responses when inquired by the health care professional. Due to the chronicity of the other ten conditions, both past and present responses were considered a 'yes' response while never response was considered a 'no' response.

To assess the within-rater or test-retest reliability of medical record data abstraction, data from half of the charts (31 of 62), selected using a random number generator (http://www.random.org), were retrieved a second time by the same abstractor (CME). Intra-class correlation coefficient was estimated to assess within-patient correlation of the abstractor's ratings to determine whether the data abstraction was consistent over time for the same rater. Inter-rater reliability was not assessed because there was only one rater (CME). Concordance between the health history information obtained from the two different sources was assessed using the kappa statistic [17]. The overall proportion of agreement (crude agreement), which is equal to the number of decisions that were agreed on by medical record and the HHQ divided by the total number of decisions available for analysis, was also calculated. To further enhance understanding of measures of agreement, positive and negative agreement measures [18] were calculated separately for the conditions with only present ('yes') and absent ('no') status. Positive agreement is the frequency at which the conditions were both noted as present by the medical record and the HHQ divided by the total frequency at which the conditions were independently indicated as present by both sources. Negative agreement is the frequency at which the conditions were both noted as absent by the medical record and the HHQ divided by the total frequency at which the conditions were independently indicated as absent by both sources. The strength of agreement of kappa values was indicated as 0 as poor; 0-0.20 as slight, 0.21-0.40 as fair, 0.41-0.60 as moderate, 0.61 to 0.80 as substantial, 0.81-1.00 as almost perfect [19].

To identify the sources of lack of agreement, discrepancies between the medical record and the HHQ were assessed. A "discrepancy" was defined as any variation in information between a patient's demographics and conditions in the medical record compared to the patient-reported demographics and conditions from the HHQ. Causes of discrepancies were classified in 1 of 3 ways (a) the condition was listed in the medical record but not endorsed by the patient's self-report; (b) the condition was endorsed by the patient but not listed in the medical record; (c) important differences observed between the patient's self-report and the information in the medical record. Important differences between the HHQ and the medical record were considered present if conflicting reports related to smoking status (never smoked, active or quit) or the status of a particular medical condition (never, present or past) were noted.

Demographic information was reported in means and standard deviation if continuous and in frequencies if categorical. Discrepancies and their sources for these 13 conditions were evaluated.

Finally, the odds ratios of agreement by age, gender, race, education and cognition as measured with the MMSE were estimated using generalized linear models with binomial distribution and logit link. Generalized Estimating Equations and empirical standard errors were used to account for the clustering of responses within persons across the 13 conditions. All analyses were done using Stata 11 statistical software [20].

## Results

Of 117 ACS patients approached for participation during the study period, 47 (40.2%) declined and 70 (59.8%) agreed to participate. Of those who agreed, 64 patients met enrollment criteria and provided informed consent to participate in the study. Two patients withdrew prior to completing the questionnaires and consequently 62 participants were included in the analyses (Table 1). Participants completed the health questionnaire an average of 1.7 ± 1.4 days after the health history was documented in the medical record. The mean age of the participants was 61.2 ± 10.4 years. The mean MMSE score was 27.0 ± 2.3. Thirty-five (56.4%) participants were men. There were 46 (74.2%) Caucasians and 14 (22.6%) African Americans. About one-third of patients reported not being high school graduates. The admission note was not available in electronic form in 9 patients, and the paper charts were reviewed to obtain information in these cases. In almost all cases, the source of information in the medical record was the resident or intern (54 of 62, 87.1%).

**Table 1:** Baseline Characteristics

**N**	62
**Age (years, mean ± SD)**	61.2 ± 10.4
**Male Sex**	35 (56.4%)
**Race/Ethnicity** **Caucasian**	46 (74.2%)
**Level of Education (by self-report)**	
**Less than high school**	5 (8.1%)
**Some high school**	16 (25.8%)
**High school graduate/GED**	20 (32.3%)
**Technical school graduate**	8 (12.9%)
**Some college**	9 (14.5%)
**College graduate**	3 (4.8%)
**Master's degree**	1 (1.6%)
**MMSE**	27.0 ± 2.3

MMSE, Mini Mental State Examination; GED, General Educational Development

Results of the analysis of degree of agreement are as noted in Table 2. Diabetes had an almost perfect agreement with a kappa statistic of 0.94, with emphysema at 0.82. There was substantial agreement in two conditions (cerebrovascular accident and hypertension). Four conditions (cocaine use, smoking, kidney disease, congestive heart failure) had moderate agreement while another four (arrhythmia, myocardial infarction, other cardiovascular diseases and bronchitis/pneumonia) had fair agreement. Angina was the only condition that had an agreement that was less than expected by chance with a kappa statistic of less than 0. For the three conditions where past, present and never status were independently assessed, angina had 5 responses where there was lack of agreement because a prior history of the condition was reported in either the medical record or the HHQ (but not both); smoking use had 22 such responses while cocaine use had 1(Table 3). Positive agreement ranged from 47.4% in bronchitis/pneumonia to 100% in diabetes while negative agreement ranged from 53.7% in myocardial infarction to 98.2% in emphysema. The intra-class correlation coefficient between the repeated ratings ranged from good (0.50 in other cardiovascular diseases) to perfect (1.00 in smoking and cerebrovascular accident) with a median of 0.94 in hypertension.

**Table 2:** Agreement of Conditions Relevant to the Care of ACS Patients

Comorbidity	Number	Yes/Yes	No/No	Yes/No	No/Yes	Crude Agreement	Positive Agreement	Negative Agreement	Kappa
**Angina^x^**	62	6	13	6	32	30.65	-	-	-0.0900

**Arrhythmia**	62	7	41	4	10	77.42	50	85.42	0.3677

**Myocardial Infarction**	62	32	11	9	10	69.35	77	53.66	0.3079

**CHF**	59	11	37	6	5	81.36	66.67	87.06	0.5374

**Hypertension**	59	41	12	3	3	89.83	93.18	80.00	0.7318

**Other CVD**	61	13	28	0	20	67.21	56.52	73.68	0.3737

**Diabetes Mellitus**	62	30	30	0	2	96.77	100	96.77	0.9356

**Bronchitis/Pneumonia**	62	9	33	19	1	67.74	47.37	76.74	0.3096

**Emphysema**	62	5	55	2	0	96.77	83.33	98.21	0.8160

**CVA**	61	8	49	0	4	93.44	80	96.08	0.7626

**CKD**	62	9	42	1	10	82.26	62.07	88.42	0.5190

**Cigarette Smoking ^x^**	45	22	1	0	0	66.67	-	-	0.4600

**Cocaine^x^**	53	4	43	3	2	88.68	-	-	0.5398

^x^Crude, positive and negative agreement (in %) not calculated for angina, smoking and cocaine use due to the presence of three categories of responses; ACS; acute coronary syndrome; CHF, congestive heart failure; CVD, cardiovascular diseases; CVA, cerebrovascular accident; CKD, chronic kidney disease; All yes and no reports are by self report first, followed by medical record documentation

**Table 3:** Description of Discrepant/Discordant Reports

Conditions	Discrepancies /Discordant Reports	Reason For Discrepancies/Discordant Reports		
		**Not reported by patient in HHQ**	**Not in Medical Record**	**Different Reports^×^**

**Angina**	43	32	6	5

**Arrhythmia**	14	10	4	-

**Myocardial Infarction**	19	10	9	-

**Congestive Heart Failure**	11	5	6	-

**Hypertension**	6	3	3	-

**Other CVD**	20	20	0	-

**Diabetes Mellitus**	2	2	0	-

**Bronchitis or Pneumonia**	20	1	19	-

**Emphysema**	2	0	2	-

**CVA**	4	4	0	-

**Chronic Kidney Disease**	11	10	1	-

**Cigarette Smoking**	22	0	0	22

**Cocaine use**	6	2	3	1

**Any Discrepancy**	180	99	53	28

^×^Discrepancies due to different reports noted only for angina, cocaine use and cigarette smoking; HHQ, Health History Questionnaire; CVD, cardiovascular diseases; CVA, cerebrovascular accident

### Sources of discordant reports

As shown in Table 3 of the 180 total discrepancies, 99 (55%) were because a condition was reported in the medical record but not by the patient in the HHQ and 53 (29.4%) because the patient reported a condition in the HHQ that did not appear in the medical record. Hence, the most common discrepancy occurred when a condition was listed in the medical record but was not endorsed by the patient in the health questionnaire. This was most commonly observed with angina (32 such discrepancies). Arrhythmia, myocardial infarction, other cardiovascular diseases and chronic kidney disease each had at least 10 discrepancies of this type.

Accounting for the correlation of responses within an individual (Table 4), the odds ratio of agreement varied significantly only by education but not by age, sex, race and cognition. When compared to those who were not high school graduates, the odds of agreement was 45% higher for those with at least some college education than those without (95% CI 1.03 to 2.01, p = 0.034). After adjusting for age, gender, race and mini-mental state examination scores, the odds ratio was only marginally significant (OR 1.42, 95% CI 1.00 to 2.01, p = 0.053).

**Table 4:** Estimated Odds Ratios of Agreement for Various Patient-Related Characteristics

Covariate	Unadjusted Model			Adjusted Model		
	
	OR	95%CI	p-value	OR	95%CI	p-value
**Age (per 1-year increment)**	0.99	0.98, 1.01	0.454	0.99	0.98, 1.01	0.342

**Gender (female vs. male)**	1.12	0.84, 1.49	0.454	1.22	0.90, 1.66	0.194

**Race (Caucasian vs. not)**	0.95	0.69, 1.32	0.772	0.95	0.68, 1.33	0.777

**MMSE (per 1-score increment)**	1.05	0.99, 1.11	0.130	1.03	0.96, 1.09	0.434

**Education**						

**Some college or above**	1.45	1.03, 2.05	0.034	1.42	1.00, 2.01	0.053

**High school graduate or GED**	1.09	0.78, 1.52	0.630	1.09	0.79, 1.52	0.589

**Less than high school or some high school (reference)**	1.00			1.00		

Generalized linear models with binomial distribution and logit link was used to estimate probability of agreement; Generalized Estimating Equation(GEE) and empirical standard errors were used to account for clustering within persons; Adjusted for age, gender, race, Mini Mental State Examination (MMSE), education; GED, General Educational Development

## Discussion

In our study, there was a considerable variability in agreement between important health conditions documented in the medical record and by the report of patients hospitalized with ACS. While most of the conditions (8 out of 13) had moderate to almost perfect agreement, 4 conditions had only fair agreement and one condition had very poor agreement. Our results add to those of Corser, et al. who documented significant discordance between the comorbidities reported by patients and the medical record of 525 ACS patients [21].

An even larger study with over 36,000 participants which compared self-reported information on cardiovascular risk factors and the same information in the medical records found substantial variability with reports of a family history of myocardial infarction having the least agreement [22]. In a recent study done in patients with heart failure, there was significant variation in patients' perceptions of their co-morbidities and habits as compared to those documented in their providers' records [23]. The highest degree of agreement in the present study was with diabetes while the least was with angina. The high agreement in diabetes may be related to patients' awareness of this condition related to self-monitoring of blood sugar and symptoms and the frequent need for oral hypoglycemic medications and insulin that have no other indication. The poor agreement found with angina may be due to the fact that the history documented in the medical record was obtained soon after presentation to the hospital while the HHQ was administered a few hours after the admission during which the patients may have received therapeutic interventions to ease their symptoms. In addition, medications used for the treatment of angina may have other indications (e.g., hypertension), and are not unambiguously related to a single health condition like oral hypoglycemics or insulin.

The agreement for myocardial infarction was only fair. The medical record and the HHQ agreed on the presence of this condition 77% of the time and agreed on its absence only 54% of the time, the lowest negative agreement value observed. Lack of agreement was most often due to patients' not mentioning this condition in the HHQ. This may point to lack of awareness of an important diagnosis by the patient when that information is known to the health care provider through diagnostic tests (e.g. echocardiography, nuclear perfusion imaging or cardiac catheterization). This lack of awareness may reflect lost opportunities to educate patients. On the other hand, it could be also be due to poor recall since it has been shown that patients may not remember details of their health events without written records [24].

More than half of the discrepancies in this study were due to lack of self-report by the participants (Table 3). This may indicate that the medical record is a more comprehensive repository of health information. It is possible that patients may not be able to reliably recall health conditions due to the stress of acute illness or the hospitalization itself, or while being treated with certain medications. Although the sources of discrepancies are unclear in many instances, and which source to actually use as the "gold standard" (i.e., the source with the most accurate and complete repository of the patient's health history) is uncertain, it is clear that discrepancies between self report and medical record are associated with a greater risk of death [25]. It is not difficult to understand why this may be particularly true for ACS patients. Early risk stratification that guides the use of several important therapies in ACS treatment involves the use of scores that are based on findings on the electrocardiogram and laboratory tests as well as information from the initial clinical history [11]. Of the three most commonly used risk scores (i.e. Thrombolysis in Myocardial Infarction (TIMI), Global Registry of Acute Coronary Events (GRACE), and the Platelet Glycoprotein IIb-IIIa in Unstable Angina: Receptor Suppression Using Integrilin Therapy (PURSUIT) scores [26-28], only GRACE [27] does not rely, at least in part, on patient self-report. The PURSUIT risk score [28], for example, is based in part on the patient's self-report of angina in the previous 6 weeks. It is therefore notable that angina was the most common source of discrepancy in the present study, highlighting the potential important health consequences in the treatment of ACS patients that may result if findings like ours are common in clinical practice. It should also be noted that although patients whose urine toxicology screens were positive for cocaine were excluded from this study, there was still only moderate agreement in reported cocaine use between the medical record and self-reported history. In 3 instances, the medical record did not document cocaine use even when it was endorsed by the patient. Lack of information on cocaine use may be of importance since beta-blockers, which are typically used to treat ACS patients, are generally thought to be contraindicated in cocaine-induced myocardial ischemia [29]. Patients' characteristics have been shown to influence accuracy of health history [30]. In our study, we found that higher educational status is associated with a higher level of agreement between the two sources of health information. This supports the findings of another study with a similar design conducted in 228 Taiwanese with hypertension and diabetes [31]. Of note, other variables such as age, gender, and race were not shown to significantly influence agreement.

An important strength of this study is that the information from the health questionnaire was obtained nearly concurrently with the documentation of the medical history, in addition to the inclusion of 13 different health conditions which are of potential relevance to the care of ACS patients. Furthermore, patients with cocaine or other recreational stimulants found on admission toxicology screen or who had dementia or delirium that may have limited their ability to provide informed consent or reliably complete questionnaires were excluded. This may have greatly reducing the chances that variations in reports are due to cognitive impairment.

Several limitations of the present study must be considered as well. With an overall participation rate of 60%, only a relatively small number of patients was studied, all of whom had a single principal diagnosis (i.e., ACS) admitted to a specialty cardiology service at a teaching hospital. Hence, the generalizability of these findings to patients hospitalized for other reasons or to outpatients is unknown. The study lacked adequate power to detect significant differences in agreement due to certain patients' characteristics. In addition, the cross-sectional nature of this study did not allow certain information to be obtained from long-term follow-up of these patients such as the effect of these discordant reports on the clinical outcomes.

## Conclusions

In this study of patients admitted with an ACS, significant discrepancies were observed between the medical record and patient self-report for 13 health conditions of importance to the care of ACS patients. Discrepancies of the type documented here may be important to the care of any patient. However, since important treatment decisions in ACS patients are made based in part on information from the initial clinical history, the findings reported here are of great potential significance for these patients in particular. There is an urgent need for research that identifies the most effective and efficient means to obtain accurate health information. This may result in improved care for ACS patients, and improve the quality and safety of patient care more broadly.

## Competing interests

The authors declare that they have no competing interests.

## Authors' contributions

CME participated in the design of the study, performed the statistical analysis, coordinated and drafted the manuscript. KC participated in the design of the study and helped to draft the manuscript. KB participated in the design of the study and helped to draft the manuscript. KF participated in the design of the study and helped to draft the manuscript. RJ participated in the design of the study and helped to draft the manuscript. GMR participated in the design of the study and helped to draft the manuscript. GY participated in the design of the study and performed the statistical analysis. RCZ conceived of the study, participated in its design, coordinated and helped to draft the manuscript. All authors read and approved the final manuscript.

## Pre-publication history

The pre-publication history for this paper can be accessed here:

http://www.biomedcentral.com/1472-6963/12/78/prepub
